# Necrology. Sir William Macleay, Kt. F. L. S., M. L. C.

**Published:** 1892-03

**Authors:** 


### NECROLOGY.

Sir William Macleay, Kt. F. L. S., M. L. C., born at Caithness, June 13, 1820, died at Sydney, N. S. W., December 7th, 1891, aged 71 years. Sir William was spoken of as "the head and heart of the Linnean Society of N. S. W." He donated to the society the hall and ground on which it stands, a valuable library, together with £35,000 for the establishment of four fellowships, and the sum of £12,000 for the establishment of a chair or lectureship in bacteriology. The removal by death of so marked a personality, of one charactered by such broad and liberal views, by such generous and lofty aims, and by extensive knowledge and wide experience, means a loss which is simply irreparable.

